# Medical Fees, No. 3

**Published:** 1845-12-01

**Authors:** 


*Medical Fees, No.* 3.—Our contemporary, the St. Louis Medical and Surgical Journal, in some remarks on this subject, indulges the following *jeau d'esprit*, which we quote, as an equipoise, to succeed the two articles we have written on the same topic. "A fee inspires the practitioner with confidence in himself, and faith in his remedies, which is almost as important as the exercise of faith on the part of the patient—An eminent English physician once remarked that he never felt of his own pulse, nor looked at his tongue in the glass, without unconsciously slipping a guinea from one pocket into the other! Let those, therefore, who wish to derive the full benefit of a physician's advice, bear in mind the necessity of a fee." This is an *argumentum ad hominem* which we should be glad to have our patients appreciate.

